# *HTT2* promotes plant thermotolerance in *Brassica rapa*

**DOI:** 10.1186/s12870-018-1346-x

**Published:** 2018-06-20

**Authors:** Jianxia Jiang, Jinjuan Bai, Shuxia Li, Xiaorong Li, Liyong Yang, Yuke He

**Affiliations:** 1 National Key Laboratory of Plant Molecular Genetics, Shanghai Institute of Plant Physiology and Ecology, Chinese Academy of Sciences, Shanghai, 200032 China; 2 Crop Breeding and Cultivation Research Institute, Shanghai Academy of Agricultural Sciences, Shanghai, 201403 China; 3 Institute of Tropical Bioscience and Biotechnology, Chinese Academy of Tropical Agricultural Sciences, Haikou, 571101 China; 4 CAS Center for Excellence in Molecular Plant Sciences, Institute of Plant Physiology and Ecology, Chinese Academy of Sciences, Shanghai, China

**Keywords:** *Brassica rapa*, Chinese cabbage, *HTT2*, *Hsf*, Thermotolerance

## Abstract

**Background:**

Numerous regulatory genes participate in plant thermotolerance. In *Arabidopsis*, *HEAT-INDUCED TAS1 TARGET2* (*HTT2*) is an important thermotolerance gene that is silenced by ta-siR255, a trans-acting siRNA. ta-siR255 is absent from heading Chinese cabbage (*Brassica rapa ssp. pekinensis*). Our previous attempt to overexpress the endogenous *BrpHTT2* gene of heading Chinese cabbage (*B. rapa* ssp. *pekinensis*) failed because of cosuppression. In theory, heading Chinese cabbage can overexpress *Arabidopsis HTT2* to improve thermotolerance in the absence of ta-siR255-mediated gene silencing and the weak potential of coexpression.

**Results:**

To test the potential application of *HTT2* in improving crop thermotolerance, we transferred *p35S::HTT2* to heading Chinese cabbage. We tested the leaf electrical conductivity, hypocotyl elongation, and survival percentage of *p35S::HTT2* plants subjected to high-temperature (38 °C) and heat-shock (46 °C) treatment. The leaf electrical conductivity of *p35S::HTT2* seedlings under high temperature decreased but did negligibly change under heat shock. The hypocotyl length of *p35S::HTT2* seedlings increased under high temperature and heat shock. The survival rate of *p35S::HTT2* seedlings increased under heat shock. *BrpHsfs,* a subset of heat-shock factor genes, were upregulated in *p35S::HTT2* plants under high-temperature and heat shock conditions. In the field, transgenic plants with *HTT2* appeared greener and formed leafy heads earlier than wild-type plants.

**Conclusions:**

Exogenous *HTT2* increased the survival rates of heat-shocked heading Chinese cabbage by promoting thermotolerance through decreasing electrical conductivity and extending hypocotyl length. Our work provides a new approach to the genetic manipulation of thermotolerance in crops through the introduction of exogenous thermotolerance genes.

**Electronic supplementary material:**

The online version of this article (10.1186/s12870-018-1346-x) contains supplementary material, which is available to authorized users.

## Background

Crop growth and yield are seriously affected by abiotic stresses, such as heat, cold, drought, waterlogging, and salinity. Among these stresses, high-temperature stress associated with global warming is one of the major threats that may result in extensive losses in global agriculture. Thus, in recent years, numerous researchers have attempted to improve the thermotolerance of crops to decrease yield losses and to ensure global food security.

Chinese cabbage (*Brassica rapa*) is one of the most high-yielding and widely planted vegetable crop. The leaves of *B. rapa* supply vital mineral nutrients, crude fiber, and vitamins. However, *B. rapa* is extremely sensitive to heat stress, especially during its reproductive stage. In recent years, drastic increases in temperature have greatly influenced the yield and quality of *B. rapa*. Therefore, increasing the heat resistance of *B. rapa* is vital for agriculture production.

Trans-acting small interfering RNAs (ta-siRNAs), a unique class of small RNAs, have been recently identified in various plant species. These ta-siRNAs are involved in posttranscriptional gene silencing and are processed as follows: First, functional miRNAs directly bind to noncoding *TAS*, the precursor RNA of ta-siRNA. The cleavage of *TAS* is triggered by the binding of miRNAs. Subsequently, double-stranded RNAs (dsRNA) are produced from the 3^′^ fragment of *TAS* through the action of RNA DEPENDENT RNA POLYMERASE6 (RDR6). Then, dsRNAs are processed into phased 21-nt ta-siRNAs by the DCL4 enzyme. SUPPRESSOR OF GENE SILENCING3 interacts and colocalizes with RDR6 in cytoplasmic granules to stabilize the cleaved transcript. Finally, novel ta-siRNAs are produced under the action of nonhomologous miRNAs [1–4]. In *Arabidopsis*, *TAS1*, *TAS2*, *TAS3*, and *TAS4* are four families of noncoding precursor genes that generate ta-siRNAs. Members of the *TAS1* family contain three loci, namely *TAS1a*, *TAS1b*, and *TAS1c*, and require miR173 to guide transcript cleavage [5]. After the cleavage of miR173, the *TAS1* transcript can form siR480(+)/siR255, siR396(+), and siR438(+).

The expression of some *TAS1*-derived siRNAs, such as siR480(+)/siR255, is responsive to various abiotic stresses. For example, these siRNAs are down-regulated under salt, dehydration, or cold stress [6, 7]. They target *HTT1* (At4g29770), *HTT2* (At5g18040), *HTT3* (At5g18065), *HTT4* (At2g29760), and *HTT5* (At1g51670) [2, 6, 8, 9]. Furthermore, the overexpression of *HTT2* could enhance the oxidative stress tolerance of transgenic plants [10]. Heat induces the expression of all five target genes of *TAS1* siR480(+)/siR255. Microarray and quantitative real-time polymerase chain reaction (qRT-PCR) analyses have demonstrated that heat remarkably induces the expression of *HTT1* and *HTT2*. The overexpression of *TAS1a* attenuates thermotolerance by increasing the accumulation of *TAS1*-siRNAs and decreasing the expression levels of *HTT* genes. By contrast, the overexpression of *rHTT1* or *rHTT2* enhances heat resistance. Moreover, several heat-stress transcription factors (*Hsf*) genes are upregulated in *rHTT*- or *rHTT2*-overexpressing *Arabidopsis* seedlings. *HTT1* mediates thermotolerance pathways through targeting by *TAS1a* and activation by HsfA1a [9].

We had previously attempted to overexpress the endogenous *BrpHTT2* gene of heading Chinese cabbage (*B. rapa* ssp. *pekinensis*) to improve the heat resistance of *B. rapa*. However, our attempt to overexpress *BrpHTT2* in heading Chinese cabbage failed because of cosuppression. In this study, we transferred an exogenous *Arabidopsis HTT2* gene, which is silenced by trans-acting siRNA ta-siR255, to heading Chinese cabbage, which lacks an endogeneous ta-siR255 biogenesis machinery. As a result, the *HTT2* gene was overexpressed in *B. rapa*, and the thermotolerance of transgenic lines increased. Thus, the *HTT2* gene of *Arabidopsis* is ideal for improving the thermotolerance of *B. rapa* through genetic manipulation.

## Methods

### Plant materials and growth conditions

Seeds of Bre, an inbred line of heading Chinese cabbage, were germinated for 1 day on moisture-absorbent paper in a plant growth chamber at 22 °C in the dark. To induce reproductive growth to obtain plants for later use in genetic transformation, the germinated seeds were transferred to a 4 °C chamber for a 25-day vernalization period. Then, the seedlings were transplanted into nutrient soil and grown in a green house at 22 °C for approximately 1 month or until the plants bloomed. The flowering plants were then used for genetic transformation.

### Gene cloning and genetic transformation

The full-length coding sequences of *HTT2* were cloned from *Arabidopsis* by using specific primers (Additional file 1: Table S1). Then, *rHTT2* (siR255-resistant versions of *HTT2*) was obtained and introduced into pCAMBIA1301 binary vectors under the control of the CaMV 35S promoter. The p35S::*HTT2* vector was transformed into heading Chinese cabbage cv Bre via the vernalization–infiltration method in accordance with previously described methods [11].

The two lines of *p35S::HTT2* transgenic plants (namely *HTT2* OE) were designated as HTT2–2 and HTT2–4. Positive HTT2 OE transgenic plants were generated for selection by germinating sterilized seeds on MS_0_ medium containing 25 mg/L hygromycin and 250 mg/L carbenicillin. Hygromycin-resistant seedlings were transplanted into nutrient soil under greenhouse conditions and were identified by using a pair of primers specific for the Hygromycin gene sequence of pCAMBIA1301 binary vectors (Additional file 1: Table S1). Homozygous lines were obtained by self-pollinating positive transgenic plants for three generations.

### Leaf electrical conductivity measurements

The seeds of HTT2–2 and HTT2–4 transgenic plants and Bre were sown on nutrient soil. At the end of August 2015, the seedlings were transplanted to the field at the Songjiang Farm Station of Shanghai Institute of Plant Physiology and Ecology. Three large and young leaves were collected from three HTT2–2 and HTT2–4 seedlings in the folding stage. Meanwhile, three leaves were collected from three Bre seedlings. Thus, each line had three biological replicates.

Electrical conductivity was measured as follows: First, a hole puncher was used to obtain 60 leaf discs from each leaf. The leaf discs were transferred to glass test tubes filled with 10 mL of double-distilled water. Each tube contained 20 leaf discs. The tubes were then capped and marked as transgenic or Bre. The number of replicates was also marked on the tubes. Second, the first group of tubes was incubated for 1 h in a 22 °C water bath, the second group was incubated for 1 h in a 38 °C water bath, and the third group was incubated for 1 h in a 46 °C water bath. Then, the tubes were fetched from the water bath, and subjected to vacuum suction until the leaf discs had sunk to the bottom of the tubes, and shaken on an oscillator for 1 h. Each temperature had three replicates. The electrical conductivity of the samples was measured and recorded as R1. The tubes were immediately recapped after measurement. Fifth, the tubes were placed for 20 min in a 100 °C water bath. The samples were cooled to 20 °C after removal from the water bath and shaken for 20 min on an oscillator. Sixth, the electrical conductivity of the samples was measured and recorded as R2. Electrical conductivity was measured in accordance with the manufacturer’s instructions (Mettler–Toledo). Relative electrical conductivity was calculated and recorded as R3 by using the following formula: R3 = (R1/R2) × 100%. Each sample had three biological replicates. Two-tailed, unpaired *t*-tests were performed for statistical analysis.

### Measurement of hypocotyl length

The seeds of Bre and HTT2–2 transgenic plants were surface-sterilized for 30 s with 75% ethanol and then for 8 min with 0.1% mercury bichloride. The seeds were washed four times with sterile distilled water and sown onto solid MS medium with 1% sucrose in petri dishes. The petri dishes were sealed with Parafilm and incubated at 22 °C in the dark. Most of the seeds germinated after 1 day and were transferred to 2 mL tubes containing 1 mL of sterile distilled water. Then, the tubes were separately incubated for 1 h in a water bath at 22 °C, 38 °C, or 46 °C. After heat treatment, the germinated seeds were sown into the bottles containing MS_0_ medium and cultivated for 5 days in an illumination incubator at 22 °C in the dark. After 5 days, the hypocotyl lengths of HTT2–2 and Bre were measured. The numbers of germinated HTT2–2 and Bre seeds under each temperature treatment exceeded 20. *t*-tests were performed to test the significance of differences between HTT2–2 and Bre plants.

### Treatment of high temperature and heat shock

The seeds of Bre and HTT2–2 transgenic plants were surface-sterilized and sown on solid MS medium with 1% sucrose in sterile bottles. Eight seeds of Bre were sown in the left part of the bottle, and eight seeds of HTT2–2 were sown in the right part of the bottle. In total, 24 seeds of Bre and 24 seeds of HTT2–2 were sown in three bottles. The seeds were cultivated at 22 °C under long-day conditions (16 h light/8 h dark). After 11 days, the seedlings of HTT2–2 and Bre exhibited vigorous growth and two true leaves. One bottle was incubated for 1 h in a 46 °C water bath, another was incubated for 2 h in a 46 °C water bath, and the third bottle was incubated for 1 h in a 22 °C water bath as the control. The phenotypes that reflected the heat resistance of Bre and HTT2–2 seedlings were observed upon the completion of the heat-shock treatment and at 1 day after treatment. The survival rates of HTT2–2 seedlings were quantified 10 days after heat shock.

### qRT-PCR

Total RNA was extracted from heat-treated samples with Trizol reagent (Invitrogen). Total RNA was treated with DNase I (Takara, Japan) to remove residual genomic DNA. A total of 3 μg of total RNA was used for first-strand cDNA synthesis for qRT-PCR with PrimeScript™ II reverse transcriptase (Takara) and oligo (dT) primers in a 20 μL reaction volume in accordance with the manufacturer’s instructions. The cDNA reaction mixture was diluted four times, and 2.5 μL of the diluted cDNA reaction mixture was used as a template in a 20 μL reaction volume. qRT-PCR reactions included the following steps. First, the samples were preincubated at 95 °C for 3 min. Next, the samples were subjected to 45 cycles of denaturation at 95 °C for 10 s, annealing at 57 °C for 20 s, and extension at 72 °C for 20 s. Finally, the samples were subjected to 61 cycles of 65 °C for 15 s. The reactions were performed in a MyiQ2 qRT-PCR detection system (Bio-Rad, www.bio-rad.com/) using iQ SYBR Green supermix (Bio-Rad). Each experiment was conducted with three biological replicates, and each sample had three technical replicates. The *ACTIN2* gene was used as the reference gene, and relative expression levels were quantified through the 2^-ΔΔCt^ method [12]. *t*-tests were performed to analyze the significance of differences. All the primers used for qRT-PCR analysis are listed in Additional file 1: Table S1.

## Results

### Sequences and expression of *HTT* genes in Chinese cabbage

**Fig. 1:**
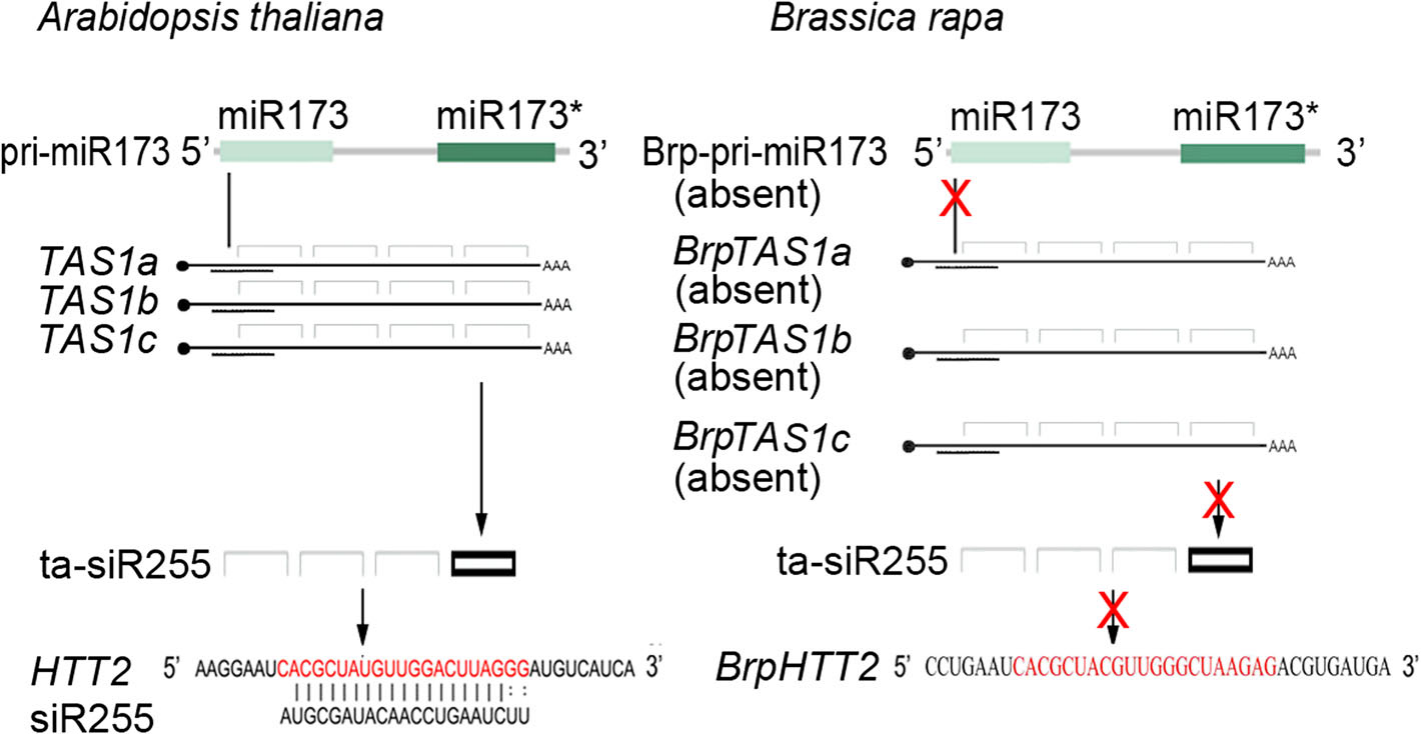
Comparison of the sequences of pri-miR173, *TAS1*, and siR255 genes from *A. thaliana* with those of their homologs from *B. rapa*

*Arabidopsis* possesses four families of noncoding precursor genes. These families include *TAS1, TAS2*, *TAS3*, and *TAS4*. Members of the *TAS1* family have three loci—*TAS1a, TAS1b*, and *TAS1c*—and require miR173 to guide transcript cleavage in ta-siRNA generation [5]. The *TAS1* loci code for multiple ta-siRNAs, including siR255, siR396(+), and siR438(+) [2, 6, 8, 13]*.* siR255 targets *HTT1*, *HTT2*, *HTT3*, *HTT4*, and *HTT5* [9]. *B. rapa* is closely related to *Arabidopsis*, and both plants are crucifers. We used the Brassica database (BRAD) website (http://brassicadb.org/brad/) [14] to search for the homologs of *MIR173*, *TAS1*, and *HTT* in *B. rapa*. We did not identify any homologs of *MIR173* and *TAS1* in *B. rapa* (Fig. 1). Moreover, we did not find siR255 from the small-RNA data of *B. rapa* [15]. The absence of *BrpMIR173*, *BrpTAS1*, and siR255 from *B. rapa* indicates that *B. rapa* lacks the siR255 biogenesis system.

We identified five homologs of *HTT* genes, namely Bra010276, Bra010277, Bra010278, Bra011210, and Bra039897 in *B. rapa*. We designated Bra010278 and Bra039897 as *BrpHTT1a* and *BrpHTT1b*, respectively. We designated Bra011210, Bra010276, and Bra010277 as *BrpHTT2*, *BrpHTT3* and *BrpHTT4*, respectively. Bra010276, Bra010277, and Bra010278 are three adjacent genes on the A08 chromosome. Bra011210 and Bra039897 are located on the A01 and A07 chromosomes, respectively. The mRNA sequence homology between Bra010276 and Bra010277 is 85.6%.

**Fig. 2:**
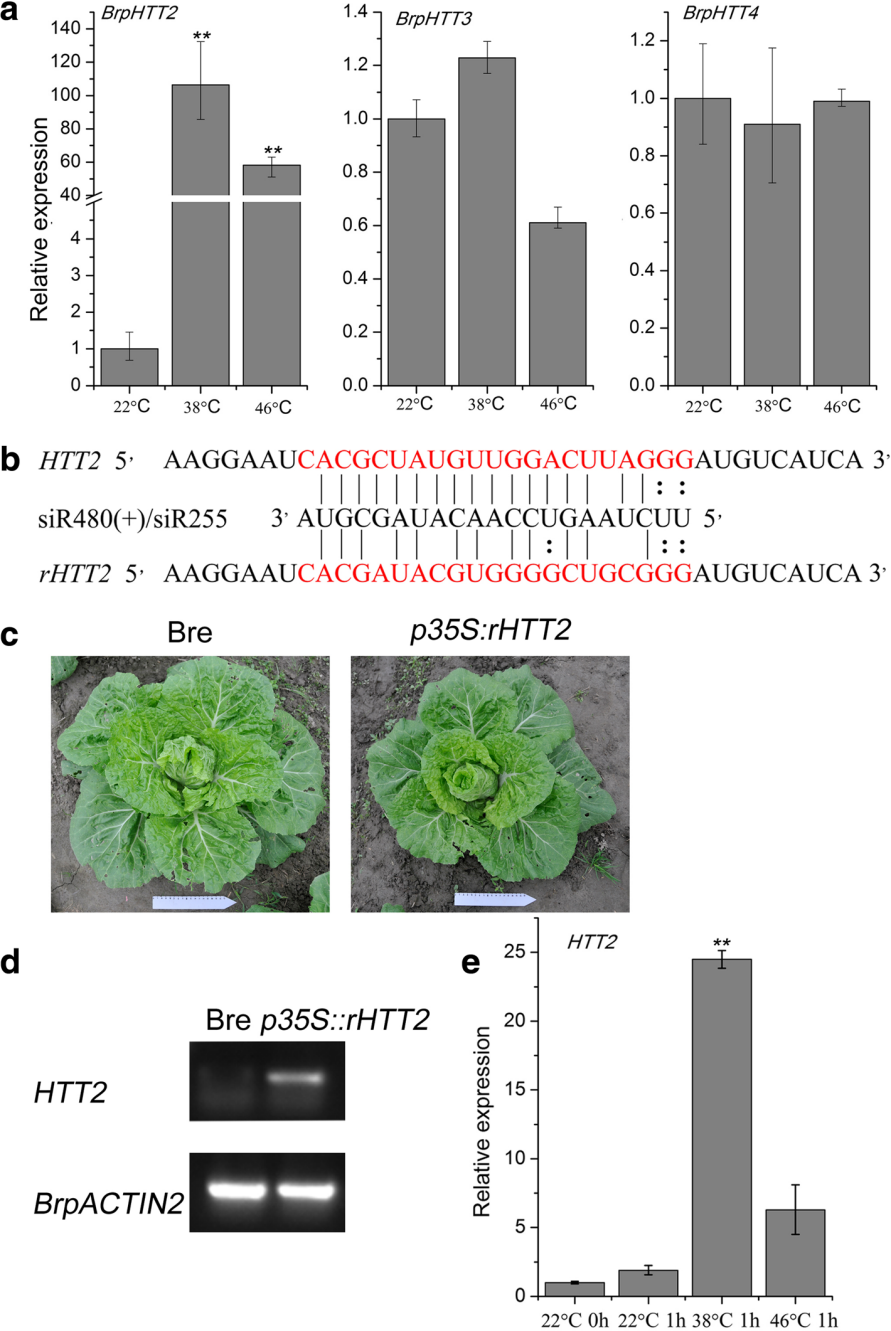
Expression levels of *BrpHTT* genes after heat-shock treatment and the phenotype of *HTT2-*overexpressing transgenic plants. (**a**) Expression levels of *BrpHTT* genes after heat-shock treatment. (**b**) rHTT2 RNA sequences complementary to siR255. (**c**) HTT2–2 and Bre plants at the folding stage. (**d**) Quantitative real-time polymerase chain reaction (qRT-PCR) results showing the relative expression levels of *HTT2* in Bre and HTT2–2 plants. (**e**) qRT-PCR results showing the relative expression of *HTT2* in HTT2–2 plants after heat treatment at different temperatures. Error bars represent standard deviation (*SD*). Differences were considered significant (*) if *p* < 0.05 (Student’s *t*-test) and highly significant (**) if *p* < 0.01 (Student’s t-test).

*HTT1* and *HTT2* genes are highly up-regulated in *A. thaliana* seedlings under heat shock [9]. We performed qRT-PCR to detect the expression levels of *BrpHTT2*, *BrpHTT3*, and *BrpHTT4* in heat-shocked Bre seedlings. The gene expression levels of *BrpHTT2* highly significantly (*p* < 0.01) increased after heat treatment and heat shock. The transcript level of *BrpHTT3* slightly increased after heat treatment at 38 °C and decreased after heat treatment at 46 °C. The expression level of *BrpHTT4* remained nearly unchanged after heat treatment (Fig. 2a). These results indicate that similar to that of *HTT2* in *Arabidopsis*, the expression of *BrpHTT2* in Bre is induced by heat shock.

### *HTT2* is overexpressed in *B. rapa*

We constructed rHTT2 (siR255-resistant versions of *Arabidopsis HTT2*) (Fig. 2b) under the control of the CAMV 35S promoter [9]. rHTT2 promotes thermotolerance in *Arabidopsis*. We transferred rHTT2 into Bre via vernalization–infiltration methods [11]. The seeds of F_1_ transgenic plants containing *p35S::rHTT2* were obtained through self-pollination, and homologous transgenic lines were selected. The transgenic plants were transferred to the field on August 2017 and 2018. Compared with the seedlings of field-grown wild-type plants, those of transgenic plants appeared greener and formed leafy heads earlier under high-temperature treatment (Fig. 2c). The results of semiquantitative RT-PCR showed that *HTT2* is overexpressed in *p35S::rHTT2* seedlings (Fig. 2d)*.*

The seedlings of transgenic lines were exposed to heat treatment at 38 °C or 46 °C for 1 h. The expression of *HTT2* in *p35S::rHTT2* seedlings exposed to 38 °C was up-regulated by ~ 20- and ~ 5-fold relative to that in *p35S::rHTT2* seedlings exposed to 22 °C (Fig. 2e) or 46 °C, respectively.

### *HTT2* decreases the relative leaf electrical conductivity of seedlings subjected to high temperatures and heat shock

**Fig. 3:**
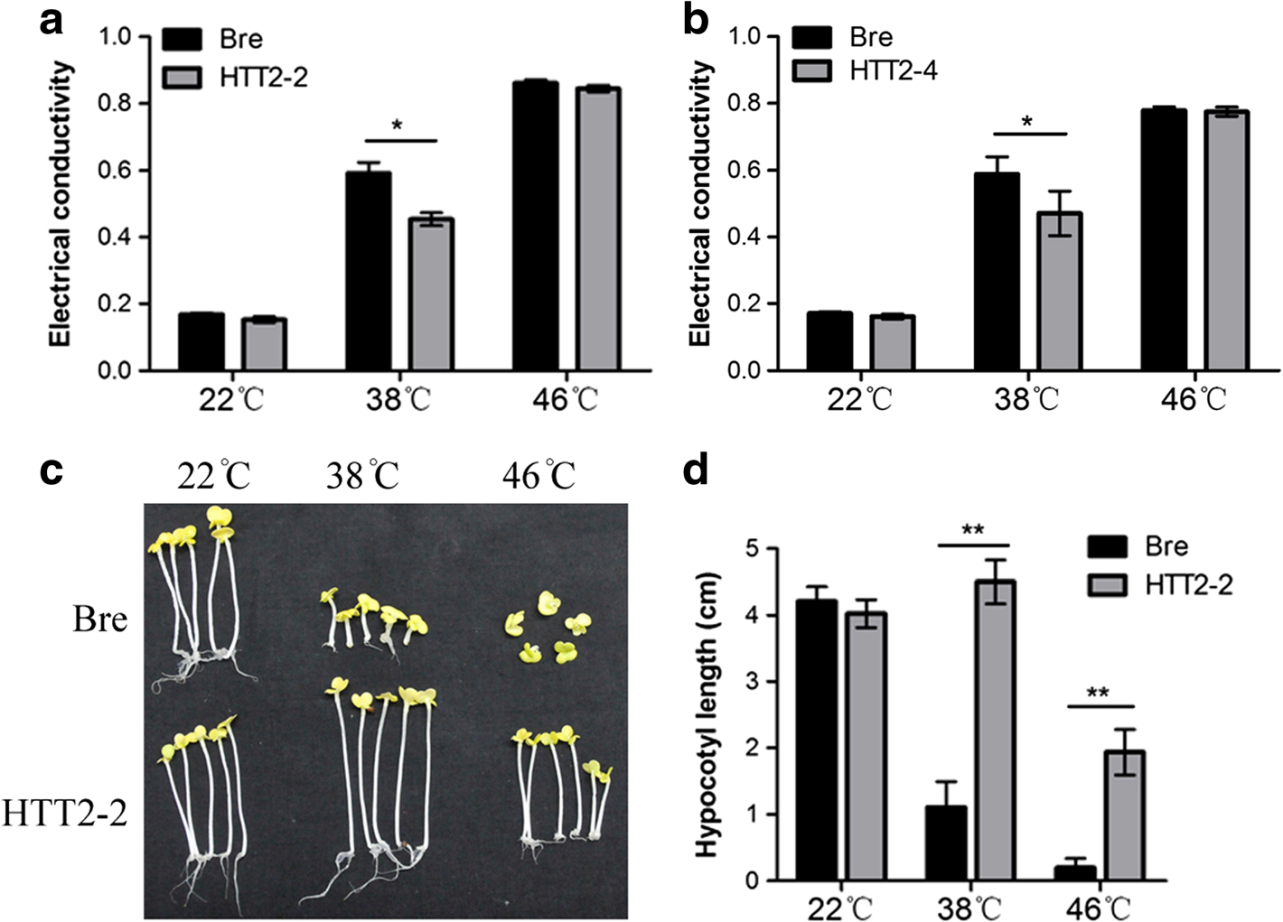
Electrical conductivity and hypocotyl length of *p35S::rHTT2* plants after high-temperature and heat-shock treatments. (**a**, **b**) Leaf electrical conductivity of HTT2–2 (**a**) and HTT2–4 (**b**) plants. (**c**) Hypocotyls of HTT2–2 plants. (**d**) Hypocotyl length of HTT2–2 plants subjected to heat treatment at different temperatures. Error bars represent *SD*. More than 20 leaves were measured for each plant of each genotype. The hypocotyl lengths of more than 20 HTT2–2 and Bre seedlings were obtained. Differences between genotypes were considered significant (*) if *p* < 0.05 (Student’s *t*-test) and highly significant (**) if *p* < 0.01 (Student’s *t*-test)

We conducted the leaf electrical conductivity test to quantify the thermotolerance of *p35S::rHTT2* plants. The two transgenic *p35S::rHTT2* lines used in this experiment were designated as HTT2–2 and HTT2–4. Leaf discs of equal sizes and numbers were collected from seedlings; immersed in water in test tubes; and incubated for 1 h in a water bath at 22 °C, 38 °C, or 46 °C. The initial electrical conductivity of the leaf discs was measured and recorded as R1. Then, the leaf discs were incubated in a water bath at 100 °C for 20 min, and the final electrical conductivity of the leaf discs was measured and recorded as R2. Relative leaf electrical conductivity was calculated and recorded as R3 by using the formula R3 = (R1/R2) × 100%. Under heat treatment at 38 °C, the relative leaf electrical conductivity values of HTT2–2 and HTT2–4 decreased relative to those of Bre (Fig. 3a, b). Unexpectedly, the relative leaf electrical conductivity values of HTT2–2 and HTT2–4 plants that had been heat shocked at 46 °C did not decrease. These results indicate that *HTT2* decreases electrical conductivity in transgenic plants exposed to 38 °C.

### *HTT2* increases the hypocotyl length of seedlings exposed to high temperature

We examined the hypocotyl elongation of the transgenic plants. The germinated seeds of HTT2–2 plants were placed in petri dishes and incubated in a water bath at 22 °C, 38 °C, and 46 °C for 1 h. Then, the germinated seeds were transferred to MS_0_ medium and grown at 22 °C in the dark for 5 days. The hypocotyl lengths of the seedlings were measured. After exposure to heat treatment and heat shock, the hypocotyl lengths of HTT2–2 plants were significantly higher than those of wild-type plants (Fig. 3c, d). The suppression of hypocotyl elongation intensified as temperature increased.

### *HTT2* enhances thermotolerance after heat shock

**Fig. 4:**
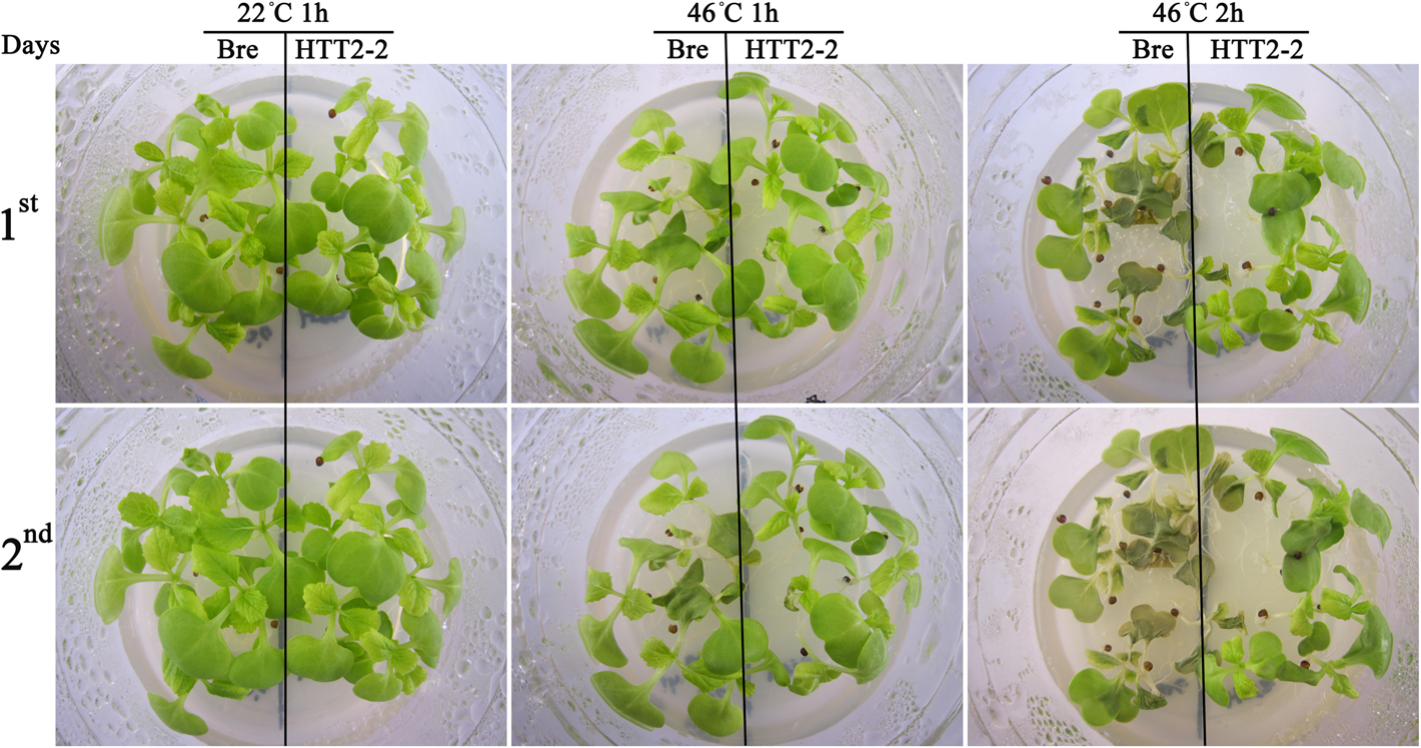
Survival rates of HTT2–2 seedlings after heat shock

The heat resistance of HTT2–2 seedlings can also be visually observed during heat treatment. Seeds were sown on MS_0_ medium. After 11 days of incubation at 22 °C under long-day conditions (16 h light/8 h dark), the seedlings were incubated in a 46 °C water bath for 1 and 2 h. After 1 h of heat-shock treatment at 46 °C, Bre seedlings withered, whereas HTT2–2 seedlings did not (Fig. 4). After 2 h of heat-shock treatment at 46 °C, Bre seedlings withered severely, whereas HTT2–2 seedlings only withered slightly. Withered Bre seedlings failed to grow and eventually died after being transferred to 22 °C for further growth. However, the HTT2–2 seedlings that only slightly withered survived, and they continued to grow after they were transferred to 22 °C. After 1 h of heat-shock treatment at 46 °C, the survival rates of Bre and HTT2–2 seedlings were 57 and 100%, respectively. After 2 h of heat-shock treatment at 46 °C, the survival rates of Bre and HTT2–2 seedlings were 0 and 100%, respectively. These results reveal that *HTT2* strongly enhances the thermotolerance of HTT2–2 seedlings.

### *HTT2* up-regulates the expression of some heat-shock factor genes

*Arabidopsis* possesses 21 *Hsf* genes that have important roles in the thermotolerance of plants. Moreover, in *Arabidopsis*, *HTT* and *Hsf* positively regulate each other, and *HTT1* mediates thermotolerance via *HsfA1a*-directed pathways [9]. We selected nine *Hsf* genes from *Arabidopsis* for further analysis. By searching for the homologous sequences of nine *Hsf* genes in *Arabidopsis*, we identified 16 homologous genes in *B. rapa*. We renamed these genes as *BrpHsf* genes (Table 1).

**Table 1:** *Hsf* genes in *Arabidopsis thaliana* and their homologs in *Brassica rapa*

*Hsf* genes in *A. thaliana*	Homologous genes in *B. rapa*		
*AtHsfA1a* (At4g17750)	*BrpHsfA1a* (Bra040179)		
*AtHsfA1b* (At5g16820)	*BrpHsfA1b-1* (Bra008593)	*BrpHsfA1b-2* (Bra023584)	
*AtHsfA1d* (At1g32330)	*BrpHsfA1d-1* (Bra023258)	*BrpHsfA1d-2* (Bra035507)	
*AtHsfA1e* (At3g02990)	*BrpHsfA1e-1* (Bra032023)	*BrpHsfA1e-2* (Bra021381)	*BrpHsfA1e-3* (Bra001071)
*AtHsfA2* (At2g26150)	*BrpHsfA2* (Bra000557)		
*AtHsfA3* (At5g03720)	*BrpHsfA3* (Bra009515)		
*AtHsfA7a* (At3g51910)	*BrpHsfA7a-1* (Bra012828)	*BrpHsfA7a-2* (Bra012829)	*BrpHsfA7a-3* (Bra033468)
*AtHsfA7b* (At3g63350)	*BrpHsfA7b* (Bra007739)		
*AtHsfB2b* (At4g11660)	*BrpHsfB2b-1* (Bra040968)	*BrpHsfB2b-2* (Bra000749)	

**Fig. 5:**
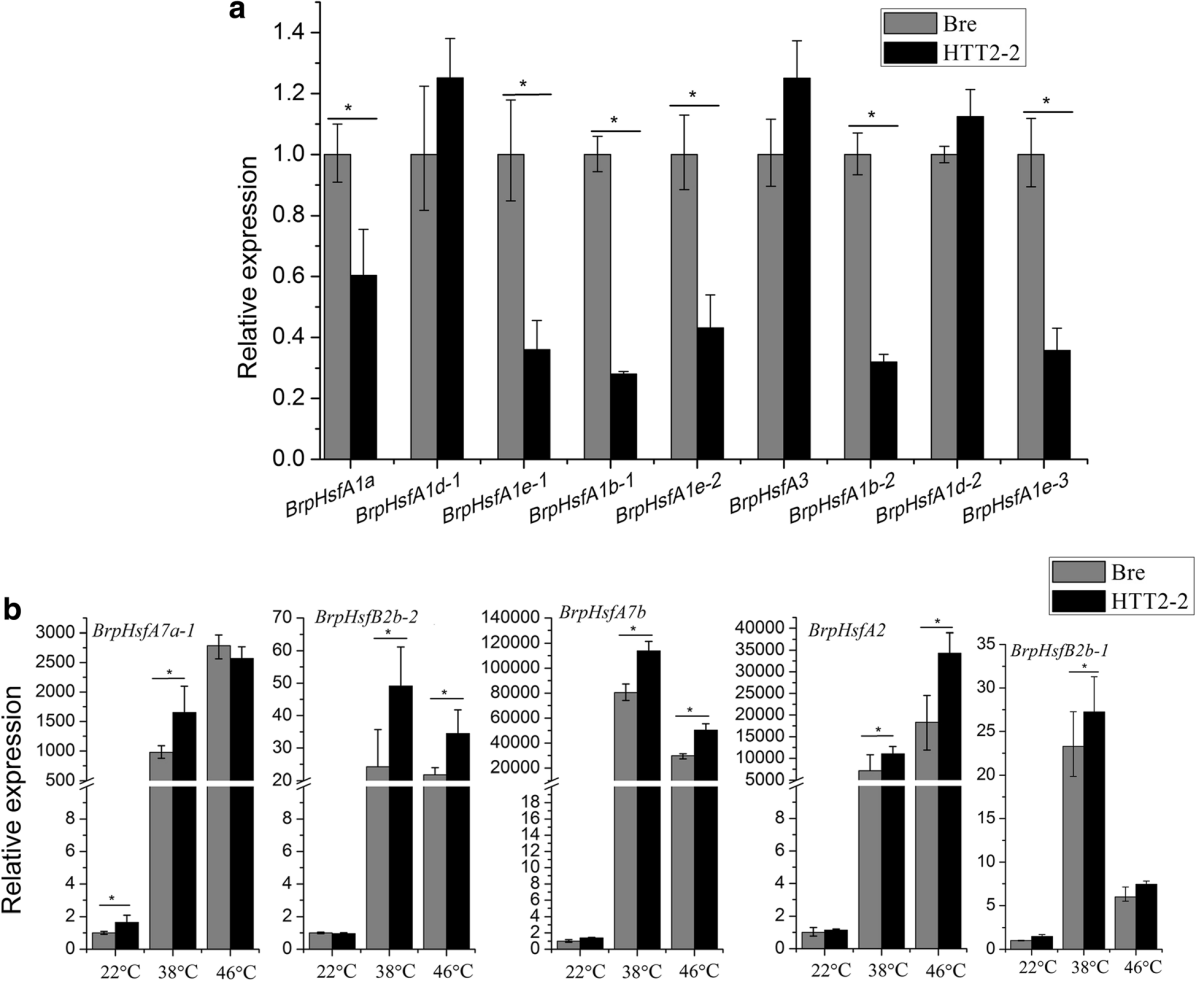
Effects of *HTT2* overexpression on the expression levels of *BrpHsf* genes. **a** Expression levels of *BrpHsf* genes in HTT2–2 seedlings under normal temperature (22 °C). **b** Expression levels of *BrpHsf* genes in HTT2–2 seedlings after heat treatment at different temperatures. Error bars represent *SD*. The relative expression abundance of each gene was measured with three biological replicates. Differences were considered significant (*) if *p* < 0.05 (Student’s *t*-test) and highly significant (**) if *p* < 0.01 (Student’s *t*-test)

To explore the roles of *Hsf* genes in heat stress, we selected 14 *BrpHsf* genes and analyzed their expression levels in the true leaves of HTT2–2 and Bre seedlings that had been subjected for 1 h to high temperature (38 °C) or heat shock (46 °C). Under normal temperature (22 °C), the expression levels of *BrpHsfA1d-1*, *BrpHsfA3*, and *BrpHsfA1d-2* were higher in HTT2–2 plants than in wild-type plants while those of *BrpHsfA1a*, *BrpHsfA1e-1*, *BrpHsfA1b-1*, *BrpHsfA1e-2, BrpHsfA1b-2,* and *BrpHsfA1e-3* were down-regulated (Fig. 5a). Notably, the expression levels of *BrpHsfA7a-1*, *BrpHsfB2b-2*, *BrpHsfA7b*, *BrpHsfA2,* and *BrpHsfB2b-1* were significantly up-regulated (*p*-value < 0.05) in HTT2–2 plants relative to that in wild-type plants after exposure to heat-shock treatment at 38 °C or 46 °C (Fig. 5b).

## Discussion

*HTT* genes are the target genes of *TAS1*-siRNAs. The expression levels of *HTT1* and *HTT2* are drastically up-regulated in *A. thaliana* seedlings in response to heat shock, whereas the expression of *TAS1a* is inhibited by heat [9]. We found that *HTT2* gene of *Arabidopsis* is highly expressed in *B. rapa*. We used qRT-PCR to analyze the expression levels of *BrpHTT2* to *BrpHTT4*. The over-expression of *HTT2* in *B. rapa* may be attributed to the following reasons: First, the *TAS1* transcripts and siR255 that act on the posttranscriptional silencing of the *HTT2* transcript are absent. Second, *HTT2* and *BrpHTT* do not cosuppress each other given their dissimilar sequences. Instead, the expression level of *HTT2* in HTT2–2 plants was up-regulated after heat treatment. In cosuppression, when a gene is introduced into a cell through transformation, neither the resident nor the transgene copy of the same gene is expressed (repeat-induced gene silencing) [16]. Apparently, *HTT2* is not cosuppressed by *BrpHTT* genes. Thus, this gene has potential applications in improving the thermotolerance of *Brassica* crops.

In HTT2–2 plants, electrical conductivity decreases after high-temperature treatment but not after heat-shock treatment, whereas hypocotyl length increases after high-temperature and heat-shock treatments, compared with those of wild-type. Hypocotyl elongation is a morphological indicator of the thermotolerance of *Brassica* crops under extremely high temperature. By contrast, electrical conductivity is a physiological indicator of heat response and thus cannot be used as an index of thermotolerance. The survival rates of heat-shocked HTT2–2 plants are considerably higher than those of heat-shocked wild-type plants. These results indicate that the over-expression of *HTT2* in *B. rapa* greatly enhances heat resistance.

A total of 21 different *Hsf* genes have been identified in *A. thaliana*. These genes are assigned to the three major classes A, B, and C on the basis of the phylogeny of their DNA binding domains and the organization of their hydrophobic repeats [17]. *HsfA1* members, which belong to group 1 in class A and include *HsfA1a*, *HsfA1b*, *HsfA1d*, and *HsfA1e*, are responsive to heat shock [18–21]. *HsfA1d* and *HsfA1e* are involved in the transcriptional regulation of *HsfA2* and function as key regulators in the *Hsf* signaling network in response to heat stress and high light [20]. *Hsf3* depresses heat-shock response and confers thermotolerance when over-expressed in transgenic plants [22]. Li et al. [9] found a regulatory relationship between *HTT* and *Hsf* genes in *Arabidopsis.* Qian et al. [23] identified a regulatory relationship between *HsfA1a* and *Hsp* genes. We detected the expression levels of 14 *BrpHsf* genes in Bre and HTT2–2 after high-temperature (38 °C) and heat-shock (46 °C) treatments. We found that the expression levels of *BrpHsfA1d-1*, *BrpHsfA3*, and *BrpHsfA1d-2* are higher in HTT2–2 plants than in wild-type plants under normal temperature conditions (22 °C). Notably, the up-regulation of *BrpHsfA7a-1*, *BrpHsfB2b-2*, *BrpHsfA7b*, *BrpHsfA2*, and *BrpHsfB2b-1* expression after either high-temperature or heat-shock treatment is more pronounced in HTT2–2 plants than in wild-type plants. The *HTT2* gene up-regulates the expression of several *BrpHsf* genes, especially under high or extremely high temperatures. We conclude that *HTT2* gene promotes plant thermotolerance by activating crucial heat-shock factor genes.

## Conclusions

Our results reveal that exogenous *HTT2* increases the survival rate of *B. rapa* seedlings under heat-shock treatment by promoting thermotolerance through reducing electrical conductivity and increasing hypocotyl length. Our work provides a new approach to the genetic manipulation of crop thermotolerance through the introduction of exogenous thermotolerance genes.

## Additional file

Additional file 1: **Table S1.** Primers for expression analysis of qRT-PCR, cDNA cloning of *HTT2*, and identification of *HPT* gene in transgenic plants. (DOCX 20 kb)

## Abbreviations

Hsf: heat-stress transcription factors
*HTT2*: *HEAT-INDUCED TAS1 TARGET2*
qRT-PCR: quantitative real-time polymerase chain reaction
RDR6: RNA-DEPENDENT RNA POLYMERASE6
*TAS*: precursor RNA of ta-siRNA
ta-siRNA: transacting small interfering RNAs
