# Supplementary material for: HTT2 promotes plant thermotolerance in Brassica rapa
Source: BMC Plant Biol. 2018 Jun 20;18:127. doi: 10.1186/s12870-018-1346-x (PMC6011422; doi:10.1186/s12870-018-1346-x)
Supplement: Supplementary file 1 — Table S1. Primers for expression analysis of qRT-PCR, cDNA cloning of HTT2, and identification of HPT gene in transgenic plants. (DOCX 20 kb) [file 12870_2018_1346_MOESM1_ESM.docx]

**Additional file 1: Table S1.** Primers for expression analysis of qRT-PCR, cDNA cloning of *HTT2*, and identification of *HPT* gene in transgenic plants.

| **Primer name** | **Sequence（5’ → 3’）** |
| --- | --- |
| *AtHTT2*-1S-EcoR1 | CGGAATTCATGAATATGATTCAGCGATTC |
| *AtHTT2*-1020A-Pst1 | AACTGCAGTTAGTTGATAGATAAGCGTGGGAT |
| *AtHTT2*-qRT-100s | GCGTCTCAGCTCAGTATTTGG |
| *AtHTT2*-qRT-312a | TCTTCTCGGTAGCCCTTTCTT |
| *BrpHsfA1a*-qRT-for | TTTTCTCGCTCCCTTCTCCC |
| *BrpHsfA1a*-qRT-rev | GCTCCTGCATCAGCACGTTC |
| *BrpHsfA1b-1*-qRT-for | CTGATGAAGCCCCAGTGAG |
| *BrpHsfA1b-1*-qRT-rev | TTCCGAAGAAAGAAACCCC |
| *BrpHsfA1d-1*-qRT-for | AATTTGCGAATGAAGGTTTCCT |
| *BrpHsfA1d-1*-qRT-rev | CCTACTACCCTCGTTTTGCTGG |
| *BrpHsfA2*-qRT-for | TGGTCGTAACAGTTTCATCGTT |
| *BrpHsfA2*-qRT-rev | TTTCAGTCTCTCTACCTCCCCT |
| *BrpHsfA3*-qRT-for | CATCAAGCACTCCCCAACAG |
| *BrpHsfA3*-qRT-rev | ATCAGGCAAACAACCACCAC |
| *BrpHsfB2b-2*-qRT-for | TAGTGGAGGAAGCAGTGGC |
| *BrpHsfB2b-2*-qRT-rev | ACGGTGAAAGAACGTGAGC |
| *BrpHsfA1e-1*-qRT-for | ATGTTTCATCCAATCCCCC |
| *BrpHsfA1e-1*-qRT-rev | TAGCTCCTCTGTGTCGCCA |
| *BrpHsfA1e-2*-qRT-for | TTCTTTTGGGAGATGTTCCG |
| *BrpHsfA1e-2*-qRT-rev | CATTCCTCAGGTTGGGTTGT |
| *BrpHsfA7a-1*-qRT-for | AGCTTCCTTTGCTTCATCATCG |
| *BrpHsfA7a-1*-qRT-rev | CAACTCTTCCCAGAAACCATCG |
| *BrpHsfA7b*-qRT-for | AGGTATTAGTTTTGTCGTGTGGG |
| *BrpHsfA7b*-qRT-rev | CTTGATGCTTTTGAGAAGGTCTC |
| *BrpHsfA1e-3*-qRT-for | CATCAGTTGGTGCTTGCGT |
| *BrpHsfA1e-3*-qRT-rev | GACTTTGGACAGCCTTCGC |
| *BrpHsfA1b-2*-qRT-for | GGAGGTGGGGAAGTTTGGAATA |
| *BrpHsfA1b-2*-qRT-rev | GCTGAGTGAGCTGAGTTGGGAG |
| *BrpHsfA1d-2*-qRT-for | CGGTTAGTTCATGTGTTGAAGTTG |
| *BrpHsfA1d-2*-qRT-rev | GGTTGTTATCAGTGGATTGTTGCT |
| *BrpHsfB2b-1*-qRT-for | CTCCTACGTGATATCCAACGCC |
| *BrpHsfB2b-1*-qRT-rev | CCTCTGAAGAACGACACCAACC |
| Bra010276-qRT-for | GTCTTCCCGCTGTCATCCTGA |
| Bra010276-qRT-rev | AAGCCCAACGTAGCTTGTTCC |
| Bra010277-qRT-for | GGAAGAAATGGCGAAACAA |
| Bra010277-qRT-rev | GAAGCATATGGGGGAACCT |
| Bra011210-qRT-for | CCGTTTGCGTCTCTCATTC |
| Bra011210-qRT-rev | CATCGTCCCGTTTCCAGTT |
| *HptII*-1S | ATGAAAAAGCCTGAACTCAC |
| *HptII*-1026A | CTATTTCTTTGCCCTCGGAC |
| *ACTIN2*-qRT-149S | CCAGAAGGATGCATATGTTGGTGA |
| *ACTIN2*-qRT-334A | GAGGAGCCTCGGTAAGAAGA |
